# Subgenomic Reporter RNA System for Detection of Alphavirus Infection in Mosquitoes

**DOI:** 10.1371/journal.pone.0084930

**Published:** 2013-12-19

**Authors:** J. Jordan Steel, Alexander W. E. Franz, Irma Sanchez-Vargas, Ken E. Olson, Brian J. Geiss

**Affiliations:** 1 Department of Microbiology, Immunology, and Pathology, Colorado State University, Fort Collins, Colorado, United States of America; 2 Department of Biochemistry and Molecular Biology, Colorado State University, Fort Collins, Colorado, United States of America; 3 Department of Veterinary Pathobiology, University of Missouri, Columbia, Missouri, United States of America; Saint Louis University, United States of America

## Abstract

Current methods for detecting real-time alphavirus (Family *Togaviridae*) infection in mosquitoes require the use of recombinant viruses engineered to express a visibly detectable reporter protein. These altered viruses expressing fluorescent proteins, usually from a duplicated viral subgenomic reporter, are effective at marking infection but tend to be attenuated due to the modification of the genome. Additionally, field strains of viruses cannot be visualized using this approach unless infectious clones can be developed to insert a reporter protein. To circumvent these issues, we have developed an insect cell-based system for detecting wild-type sindbis virus infection that uses a virus inducible promoter to express a fluorescent reporter gene only upon active virus infection. We have developed an insect expression system that produces sindbis virus minigenomes containing a subgenomic promoter sequence, which produces a translatable RNA species only when infectious virus is present and providing viral replication proteins. This subgenomic reporter RNA system is able to detect wild-type Sindbis infection in cultured mosquito cells. The detection system is relatively species specific and only detects closely related viruses, but can detect low levels of alphavirus specific replication early during infection. A chikungunya virus detection system was also developed that specifically detects chikungunya virus infection. Transgenic *Aedes aegypti* mosquito families were established that constitutively express the sindbis virus reporter RNA and were found to only express fluorescent proteins during virus infection. This virus inducible reporter system demonstrates a novel approach for detecting non-recombinant virus infection in mosquito cell culture and in live transgenic mosquitoes.

## Introduction

Alphaviruses are mosquito-borne pathogens that can cause severe human and veterinary disease, several of which are considered potential biological weapons [2,3]. Alphaviruses are a major global health concern due to the widespread prevalence of arthropod vectors and limited prevention and treatment options for infection [4]. Alphavirus infection results in a wide range of clinical symptoms, including fatal encephalitis or long-term arthritis [2,4,5].

Defining how alphaviruses infect the mosquito vector and transmit to mammalian hosts is an active area of study, but the tools for monitoring alphavirus infection in mosquitoes have largely relied on postmortem analysis or using recombinant viruses engineered to express fluorescent or luminescent proteins from duplicated subgenomic promoters (SGP) [6]. Although recombinant alphaviruses are useful tools in various applications, this approach requires that an infectious clone of the particular strain of virus be available and that the clone be engineered to express a reporter protein suitable for use in the study. Another complicating factor is that modifications to the viral genome can increase the viral genome size by over 10% and often leads to a reduction in viral replication kinetics that attenuates virulence in the mammalian and/or arthropod hosts [1,6-8] . These issues demonstrate that alternative approaches for detection of wild-type alphavirus infection in mosquito cells need to be developed to provide more physiologically relevant data. A system that would allow live visual detection of SINV infection in the mosquito vector would be a valuable tool for further understanding the transmission and infection of alphaviruses. 

Alphaviruses (Family *Togaviridae, genus alphavirus*) are positive strand RNA viruses with a genome size of ~12Kb containing a 5’ RNA cap and a 3’ polyadenylated tail [9]. Sindbis virus is considered a prototypical alphavirus and has been used extensively to understand alphavirus replication. The 5’ two-thirds of the genome encodes a polyprotein of nonstructural proteins (nsP) 1 through 4 that are required for viral RNA replication. The nsP1-4 polyprotein is initially translated from the viral genomic RNA to form the nsP1-2-3 / nsP4 complex that produces a negative strand copy of the genomic RNA. The nonstructural polyprotein is cleaved to nsp1/nsP2/nsP3/nsP4 to form the positive strand replicase complex, which produces new genomic RNAs. The positive strand replicase complex also produces 26S RNAs from a subgenomic promoter present on the negative strand RNA later in infection [9]. The structural proteins are translated from the 26S subgenomic RNA to express capsid, E2, and E1 glycoproteins that form virus particles. 

Production of the subgenomic 26S RNA is dependent on the viral replicase complex binding to the 3’ end of the viral RNA and synthesizing a negative sense RNA [9] . The requirement for the viral nonstructural protein replication complex to express proteins encoded on the subgenomic RNA provides a mechanism for expressing foreign genes only during infection (when the replication complex is present). By inserting the subgenomic promoter sequence upstream of a reporter protein (such as fluorescent proteins or luciferase enzymes), the reporter will only be expressed when virus is actively replicating within a cell, providing the replication complex *in trans*. The alphavirus subgenomic promoter has been used in alphavirus expression systems, which utilize a duplicated subgenomic promoter to express a gene of interest concurrent with virus replication [6,7,10]. These double subgenomic recombinant alphaviruses are efficient at expressing reporters, but because the reporter is inserted directly into the viral genome, this approach is limited to virus strains with infectious clones and applies an extra genetic load to the recombinant virus replication. Instead of inserting the reporter into the viral genome (11.7 kb), we inserted the reporter into the mosquito genome (1.38 billion bp), which applies a reduced genetic burden and allows detection of unlabeled, non-recombinant wild-type viruses [11]. Inserting the reporter RNA into the cell genome instead of the viral genome allows mosquito cells to express the reporter RNA constitutively, and upon virus infection, the subgenomic RNA can be synthesized and the reporter protein translated. Olivo et al previously used a similar system to express a luciferase protein in BHK cells during Sindbis virus infection; however, their system was not designed for mosquito cells and the use of luciferase did not allow for visual detection of real-time infection [12-14]. Alphaviruses are transmitted mainly by mosquito vectors and better tools are needed to monitor transmission between mosquitoes and vertebrate hosts. We have adapted the system described by Olivo et al to function in mosquito cells. Although the luciferase reporter functioned well in BHK cell culture, we sought to develop a system that would produce fluorescence within mosquitoes when infected by alphaviruses. A fluorescent reporter was used instead of the luciferase to provide real-time visual detection and avoid the difficulty of injecting mosquitoes with luciferin in order to detect the luciferase reporter. The fluorescent reporter protein provides a convenient way to monitor infection as it progresses through the mosquito.

To visually track alphavirus infection in mosquitoes, we used insect specific promoters to constitutively transcribe reporter RNA constructs in mosquito cells. The reporter RNAs can be replicated by transcomplementing viral proteins and produce a fluorescent reporter protein only during infection. Here we show the ability of our subgenomic reporter constructs to detect alphavirus infection in mosquito cell culture and in transgenic mosquitoes. These results represent the first time a replication-competent alphavirus RNA has been launched from DNA in mosquito cells and demonstrates a new method for detecting alphavirus infection in mosquitoes. 

## Materials and Method

### Plasmid Constructs

Reporter RNA constructs were engineered with the baculovirus immediate early promoter (IE3) [15] for transcription in C6/36 cell culture and the *Ae. aegypti* poly-ubiquitin (PUb) promoter [16] to transcribe the reporter RNA in transformed *Ae. aegypti* mosquitoes. The reporter constructs were developed from SINV sequences using the TE3’2J/TR339 strain of Sindbis and from SINV replicon pBG254 and pBG60 previously described [17,18]. The 5’ and 3’ UTR sequences were included from SINV (5’ end to the start of the nonstructural protein and the 3’ end from the c-terminus of E1 through the poly A tail). The first 143 residues of nsP1 were inserted in frame with an enhanced Green fluorescent protein (eGFP) gene followed by stop codons used to identify transfected cells and stop translation initiated from the 5’ end of the reporter RNA. We then inserted the subgenomic promoter sequence followed by the mCherry gene, which will produce a subgenomic RNA that mCherry can be translated from in the presence of transcomplemented nsP1-4 proteins. 50 adenosine residues were added downstream of the 3’ UTR to produce a polyadenylated end (pBG426) as previously described [19]. 

eGFP was removed from pBG426 by BglII restriction enzyme digest and the plasmid was re-ligated to produce plasmid pBG446. Alternatively, eGFP was removed from pBG426 with BglII and replaced with the antibiotic resistance gene puromycin acetyltransferase (PAC) [20], which had been amplified with primers containing BglII sites (BG661- ATGCAGATCTTTCGTGAAGACCC and BG662-CCTGAGATCTGGCACCGGGCTTGC). The PAC gene was ligated into the BglII site, producing plasmid pBG461. 

pBG460 was designed using the West African strain of Chikungunya virus (CHIKV) 37997(pCHIK-37997–5GFP, GenBank accession number EU224271) [21]. The CHIKV sequence included identical regions (5’ UTR, nsP1, SGP) as the SINV sequence (pBG426) and was synthesized by GenScript. The CHIKV reporter sequence was amplified from the synthesized plasmid using a reverse primer with a 5’ end XhoI site and a forward primer with the 5’ end having 20bp overlap with the 3’ end of the IE promoter (BG657-GTTCATGTTGGATATTGTTTCATGGCTGCGTGAGACACACG and BG658-CGGGCCCTCAAGACTCGAG ). The IE promoter was amplified with a forward primer containing a 5’ NheI site and reverse primer with 5’ overlap of the 5’ CHIKV UTR (BG655-GTCGGGTCCATTGTCCGTGTG and BG656- CGTGTGTCTCACGCAGCCATGAAACAATATCCAACATGAAC). The two PCR products were the templates for fusion PCR for 10 rounds followed by addition of primers BG655 and BG658 for 30 more cycles. The resulting full-length fragment was ligated into the NheI and XhoI sites in pBG426.

Additional subgenomic promoters from Western equine encephalitis virus (WEEV)) (pBG447) or CHIKV (pBG448) were inserted into pBG446 by ligating a virus specific SGP/mCherry PCR product into the XbaI site. The WEEV and CHIKV SGPs were amplified with forward primers containing 5’ XbaI sites (BG582- TACATCTAGACTCTACGGCTGACCTAAATAGG and BG581- TACATCTAGACTGTACGGTGGTCCTAAATAGG). The reverse primer annealed to the 3’ end of mCherry and had a 5’ XbaI site (BG576- ATATTCTAGACTACTTGTACAGCTCGTCCATGC).

 The IE3 promoter was replaced with the *Ae. aegypti* poly ubiquitin promoter (PUb) [16] and inserted into the transposon backbone containing the 3xP3 promoter and an eGFP gene for mosquito transgenesis [22]. The IE3 promoter was replaced with the PUb promoter through overlapping PCR amplification. The SINV reporter construct was amplified with a forward primer containing 5’ 19bp overlap with the PUb and a reverse primer with a terminal AscI site (BG 671- GCAAAGGCAAAACCAGCTCATTGACGGCGTAGTACACAC and BG 675-CTGGCGCGCCGCCCTCAAGACTCGAG ). The PUb fragment was amplified with a forward primer containing a 5’ AscI site and a reverse primer with 5’ 20bp overlap with the beginning of the reporter construct. (BG669- CTGGCGCGCCTATCTTTACATGTAGC and BG670- GTGTGTACTACGCCGTCAATGAGCTGGTTTTGCCTTTGC). The two PCR fragments were fused for 10 rounds, then primers BG669 and BG675 were added to amplify the fusion PCR product. The PCR product and destination plasmid were digested with AscI and ligated into the transposon backbone (pMos[3xP3-eGFPaf]) resulting in pBG471 [22]. All clones were verified with sequencing. 

### Cell Culture, Transfection, and Viruses

C6/36 *Aedes albopictus* cells were grown in 6-well cell culture plates with L-15 media containing 10% FBS, 100U/ml penicillin/streptomycin, and 5% NaHCO3. Cells were maintained in a 28°C incubator. C6/36 cells were transfected with Mirus 293T transfection reagent following manufacturer’s protocols for 1µg DNA. 12 hours post transfection; the cells were infected with SINV at an MOI of 10. At 36-48 hours post infection, the cells were examined for mCherry expression. Stable reporter expressing cells were selected with 2µg/ml of Puromycin and drug was replaced every 3-4 days. 

TE3’2J SINV virus stocks for *in vitro* infections were produced from plasmid transfection into Baby Hamster Kidney (BHK) cells [8] . MRE16 5’ds GFP and MRE16 5’ds were generated from infectious clones [23,24]. Chikungunya virus (La Reunion strain LR2006-OPY-1) and Western Equine Encephalitis virus (McMillian strain) infections were performed in biosafety level 3 [25,26]. The CHIKV LR strain was used because we had access to an infectious clone containing a 5’ duplicated subgenomic promoter and GFP gene for visual detection of infection. Sindbis and West Nile virus (Kunjin subtype) (KUNV) infections were performed at biosafety level 2 [27]. 24 hours after plating cells or 12 hours post transfection, cells were infected by replacing media and adding in sufficient amounts of virus for desired MOI. 

### Fluorescence quantification

Fluorescent images were acquired with a Nikon Diaphot 200 inverted fluorescent microscope. Images were analyzed and fluorescence was quantified using Image J software [28,29]. Specifically, images were separated to RGB color channels. The red or green stack was selected and the threshold was adjusted to detect only fluorescent cells that were brighter than negative controls. Following the threshold adjustment, fluorescent cells were analyzed for pixel counts, total area, average size, area fraction, and integrated density. Relative fluorescence corresponds to integrated density (intDen) value. Fold change in fluorescence was determined by dividing intDen values of infected images by the uninfected control. 

### RNA and Protein quantification

RNA was extracted using Trizol reagent as previously described [30] . Northern Blots to detect RNAs were performed with DIG-labeled RNA probes (Roche DIG Northern kit) specific for the mCherry gene in the reporter and subgenomic RNA [31]. qRT-PCR was performed using the Brilliant III Ultra-Fast SYBR Green QPCR kit (Stratagene, Agilent Technologies) with primers corresponding to the mCherry gene. Total protein was extracted from triturated mosquitoes in PBS. Primary rabbit anti-GFP antibody (Cat#ab290, Abcam, Cambridge, MA) or mouse anti-mCherry antibody (ab125096, Abcam, Cambridge, MA) were used to detect GFP and mCherry in western blot assays. Secondary goat polyclonal antibodies conjugated to horseradish peroxidase that are anti-rabbit (anti-rabbit, Cat#ab97051; anti-mouse; Cat#ab97023 Abcam, Cambridge, MA) were used to detect primary antibodies. Northern and Western blots were imaged using a Chemidoc XRS for chemiluminescent detection with HRP peroxide/luminol (Thermo-Scientific, Rockford IL) and CDP-Star (Sigma-Aldrich, St. Louis MO). 

### *Aedes aegypti* transformation

*Aedes aegypti* mosquitoes from the Higgs White Eye Strain (HWE) [32] were hatched and allowed to mature to adulthood. Females were given a bloodmeal consisting of defibrinated sheep’s blood (Colorado Serum Company. Denver, CO) 4 days before oviposition. On the day of injection, females were presented with an oviposition paper inside a 50ml conical tube, and eggs were collected for injection [33]. 1,736 eggs were injected at the posterior pole with plasmid pBG471 (Mariner Mos1 and reporter construct) and a helper plasmid containing the mariner transposase gene using an Eppendorf FemptoJet injector [34]. Following injection, eggs were returned to oviposition paper for 4-5 days to mature. The 327-hatched larvae (18% survival) were grown and separated into individual containers as pupae. The emerged adults were pooled together into 65 families depending on gender (1 male with 15 HWE wild-type (wt) females, or 12 female with 3 HWE wt males). Each of the 65 families was bloodfed three times and eggs were collected. The eggs were hatched and screened for eGFP expression in the eyes of the larvae on a fluorescent dissecting microscope. Nine families contained positive larvae and were outcrossed with HWE for 3 generations then were intercrossed for subsequent generations. Two families did not produce viable offspring, but the remaining 7 lines were stable through at least 10 generations. Each generation is screened for positive GFP eye expression as a marker for the presence of the transposon. 

### Mosquito infections

7-14 day old mosquitoes were orally infected with a blood meal containing GFP-expressing SINV (MRE16 5’dsGFP) [8]. Virus for bloodfeeds was prepared by infecting Vero cells with the respective virus 36-48 hours prior to bloodfeeds [35,36]. The virus was collected prior to feeding by scraping cells, centrifuging supernatant, removing media to desired volume (5mls) and then mixed with 5 ml sheep’s blood and 1 ml ATP [37]. Mosquitoes were fed through an artificial glass feeder with hog’s gut as the membrane. Bloodfed mosquitoes were separated and maintained following the feeding and the bloodmeal was titered to verify virus titers. Mosquitoes engorged with a bloodmeal were assumed infected, and at 7dpi and 14dpi the mosquitoes were screened for GFP (infection) and mCherry (reporter of infection) using a dissecting fluorescence microscope. Midguts were dissected from whole mosquitoes to examine reporter protein expression within internal tissues. Alternatively, whole mosquitoes were triturated, supernatant was filtered through 0.2uM syringe filter, and samples were assayed for virus titers by plaque assay [30]. 

## Results

### Rationale for design of Sindbis virus subgenomic reporter constructs

**Figure 1:**
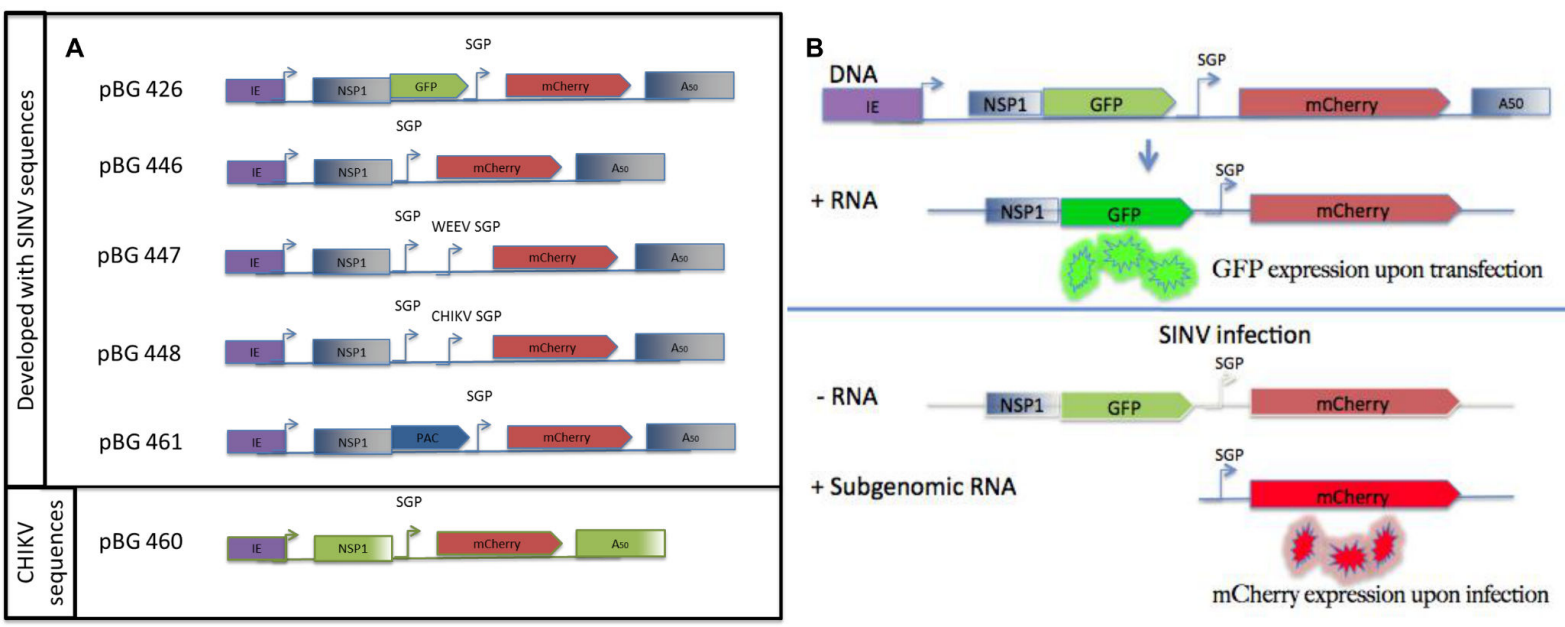
Diagram of Subgenomic reporter RNA constructs. **A**) Transcription of each reporter RNA is initiated from the baculovirus IE promoter (IE), with 5’ UTR, 3’ UTR, and subgenomic promoter sequences derived from SINV (pBG426, 446, 447, 448, and 461) or CHIKV (pBG460). All constructs include the first 143 residues of nsP1 to provide conserved RNA sequence elements and 50 adenosines at the 3’ terminus. pBG426 encodes eGFP fused to nsP1 for detection of transfected cells expressing the reporter RNA. pBG446 does not contain GFP and is a simplified construct compared to pBG426. pBG447 and 448 have an additional virus specific (WEEV or CHIKV) subgenomic promoter inserted downstream of the SINV SGP. pBG461 contains the puromycin acetyltransferase gene fused to nsP1 for selection in stable cell lines. pBG460 is a CHIKV specific reporter construct engineered directly from CHIKV sequences. **B**) Diagram of reporter RNA replication and mCherry expression.

**Figure 2:**
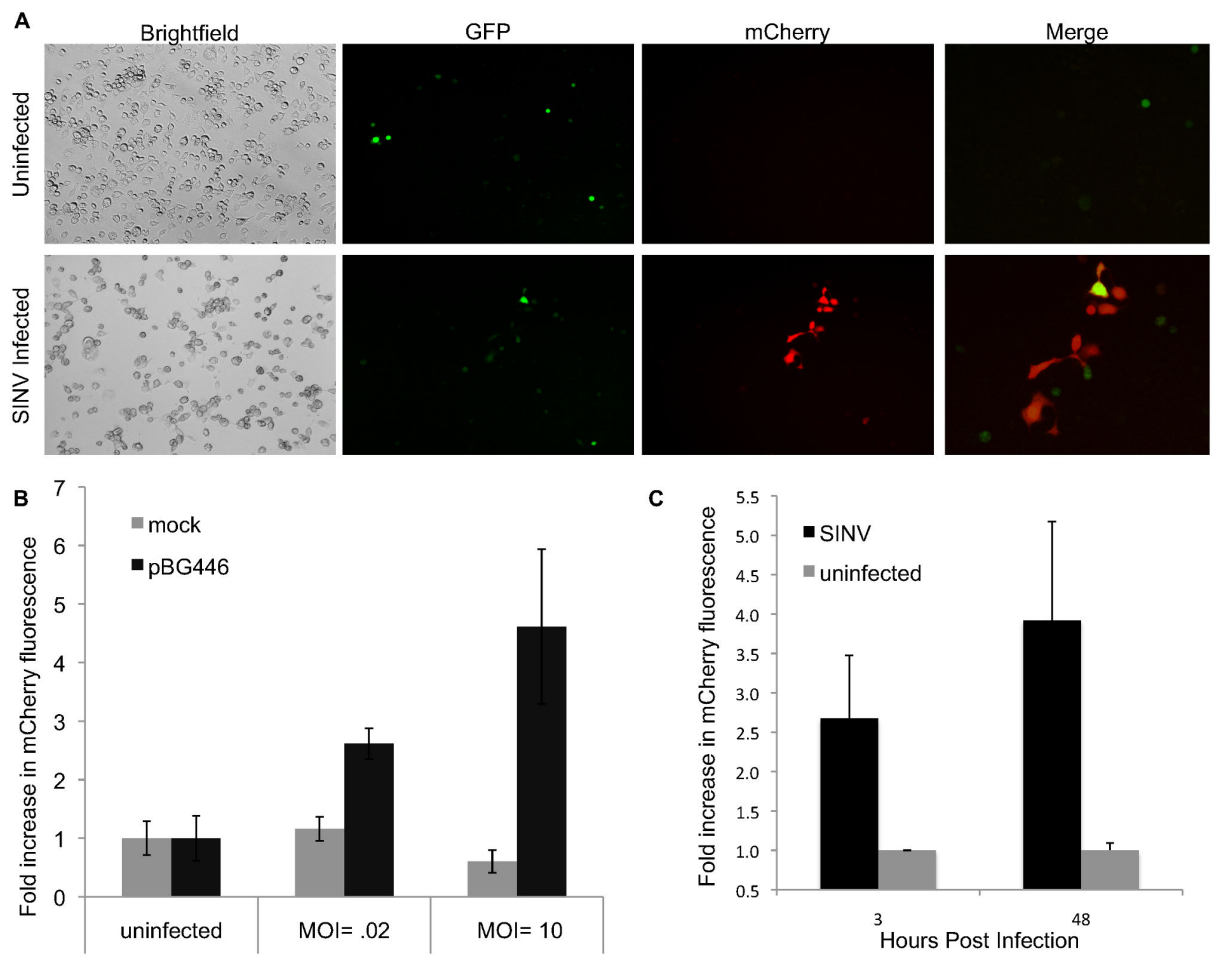
Expression of Alphavirus Reporter RNAs in SINV Infected Cells results in mCherry Production. C6/36 cells were transfected with pBG426 and subsequently infected with pBG167 TE3’2J SINV for 36 hrs. **A**) Brightfield, GFP, and mCherry fluorescence was determined for each sample. Uninfected (top) and infected (bottom). **B**) Fluorescence increase with different MOIs of SINV. C6/36 cells were transfected with pBG446 and infected for 36 hrs at the indicated MOI. Images were collected from each experimental well and the mCherry fluorescence was quantified. **C**) Increased fluorescence with increased infection times. C6/36 cells were transfected with pBG446 and infected at MOI = 10. Images were collected at the indicated times and fluorescence quantified.

Sindbis virus infection was detected by utilizing the viral subgenomic promoter to induce synthesis of a subgenomic RNA expressing mCherry only during active virus replication. Plasmids were engineered to contain alphavirus RNA elements (5’ UTR, SGP, 3’ UTR) with reporter proteins replacing the viral proteins. The 5’ end of the reporter genome was aligned with the transcription start site of the DNA pol II baculovirus IE promoter to allow full-length reporter RNAs to be transcribed in mosquito cells [8] . The 5’ and 3’ UTRs were maintained to allow the reporter RNA to be replicated in the presence of viral non-structural proteins [38,39]. The nonstructural proteins were removed from the 5’ open reading frame, except for the first 143 amino acids of nsP1 which contain conserved sequence elements required for RNA replication [9] (Figure 1). Initial constructs contained an eGFP gene fused to the nsP1 fragment that allowed for visual detection of cells expressing the full-length reporter RNA (Figure 1 and 2). The structural proteins, which are encoded from a subgenomic RNA at the 3’ end of the genome, were replaced with a mCherry fluorescent protein under control of the subgenomic promoter. The IE promoter drives expression of the reporter RNA in transfected cells which was evident by visual detection of eGFP expression (Figure 2A). Open reading frames located at the 3’ end of the genome cannot be translated from the full length reporter RNA due to stop codons upstream of the subgenomic promoter. Transcription from the subgenomic promoter, which is initiated by the nonstructural protein replication complex, generates a short subgenomic RNA that is competent for mCherry translation. The full-length reporter RNAs do not contain the open reading frames for the nonstructural proteins, so the reporter RNA can only be replicated and produce a subgenomic RNA only when replication competent virus infects the cell and provides the nsP1-4 proteins *in trans*. The infecting virus translates its own nonstructural replication complexes, which bind to the full-length reporter RNAs already present in the cell and replicates the reporter RNA. The subgenomic RNA is transcribed from the negative strand copy of the replicating full-length reporter RNA, and the fluorescent mCherry protein is translated from the newly synthesized subgenomic RNA (Figure 1B). 

### Reporter RNA constructs can detect infection in mosquito cells

Reporter RNA expressing plasmids transiently transfected into *Aedes albopictus* C6/36 cells show eGFP expression within 6 hours after transfection, indicating transcription of the reporter RNA is occurring within the transfected cells. The low number of eGFP positive cells reflects the low transfection efficiencies we commonly observe with C6/36 cell transfection. mCherry expression is not observed in uninfected cells, whereas mCherry fluorescence can be observed following infection with Sindbis virus (Figure 2A). Interestingly, we observed that cells that strongly expressed eGFP tended to have reduced mCherry expression upon infection, and conversely observed that cells expressing low levels of eGFP tended to display higher levels of mCherry following infection. mCherry fluorescence was quantified from images collected at given time points and multiplicity of infections. Significant expression of the reporter was detected with a virus MOI of as little as 0.02 at 36 hrs post infection and infection with an MOI of 10 provided significant mCherry fluorescence at 3 hours post infection (Figure 2B and 2C). These results indicate that infectious Sindbis virus can provide the replication complex *in trans* to activate the subgenomic promoter on reporter RNAs and express subgenomic RNA encoded proteins. We removed the eGFP to simplify the system (pBG446), and consistently observed mCherry expression during SINV infection but not in the absence of SINV (data not shown). Therefore, this system is able to visually detect SINV infection in cultured mosquito cells. 

### Reporter RNA expression is virus specific

**Figure 3:**
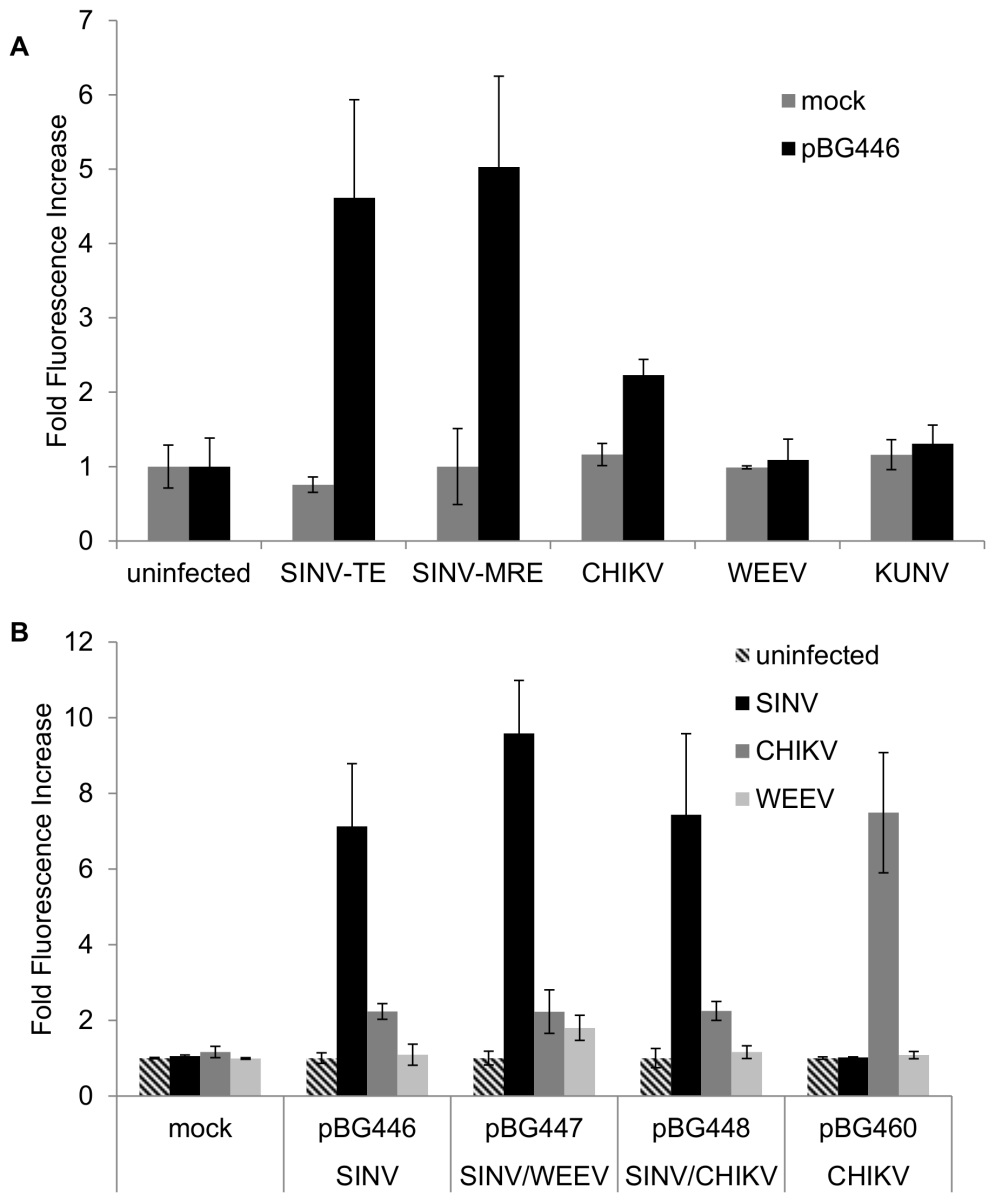
Sindbis subgenomic reporter detects infection with different SINV strains and similar old world alphaviruses, but not new world alphavirus or flavivirus infections. **A**) C6/36 cells were transfected with pBG446 and then infected with different viruses (SINV (TE3’2J strain), SINV (MRE16 strain), CHIKV (La Reunion strain), WEEV (McMillan strain), and West Nile (Kunjin subtype). **B**) Virus-specific subgenomic promoter constructs for WEEV and CHIKV (pBG447 and pBG448), pBG446 (SINV specific), and pBG460 (CHIKV specific) were transfected into C6/36 cells and subsequently infected with SINV, CHIKV, WEEV, or uninfected mock control. All Images were taken at 36hrs post infection and mCherry fluorescence was quantified.

To determine if activation of the subgenomic promoter on the reporter constructs is virus specific, we tested if related alphaviruses or unrelated flaviviruses could activate the Sindbis virus subgenomic promoter. C6/36 cells were transfected with pBG446 and infected with different viruses. mCherry expression was visually detected by fluorescence microscopy at 36 hours post infection (MOI 10) and fluorescence intensity was calculated. Infection with two strains of SINV (TE and MRE) resulted in a significant increase in mCherry expression, indicating that the detection was not strain specific (Figure 3A). However, infection with a flavivirus (KUNV) or a New World alphavirus (WEEV) [40] did not result in detectible mCherry expression (Figure 3A). Old World Chikungunya virus (CHIKV) was able to induce expression of mCherry, although to a lesser extent than Sindbis virus infection. This confirms that the viral replication complex has specificity for binding to the reporter RNA sequences, likely the 5’ or 3’ UTR in conjunction with the SGP, and that some cross-reactivity can be observed between related alphaviruses [38,39]. 

### The subgenomic promoter is not sufficient to induce subgenomic RNA synthesis

To determine if this system could detect a broad range of alphaviruses, an additional subgenomic promoter was inserted behind the SINV SGP. Theoretically, a reporter system with multiple SGPs from different viruses would be able to detect infection of any of the viruses. The WEEV or CHIKV subgenomic promoter sequence was added 3’ to the SINV subgenomic promoter in the SINV construct (pBG447 and pBG448- Figure 1). C6/36 cells transfected with pBG447 or pBG448 and infected with WEEV or CHIKV respectively, did not result in significant expression of mCherry (Figure 3B). However, a construct that was engineered and developed entirely based on CHIKV sequences showed detectable amounts of mCherry fluorescence during CHIKV infection but not with other viruses (Figure 3B). These results indicate that virus specific 5’ UTR, 3’ UTR, and subgenomic promoters are all required for the production of the 26S RNA from the reporter RNA and subsequent protein expression. 

### Stable cell line expressing reporter RNA

**Figure 4:**
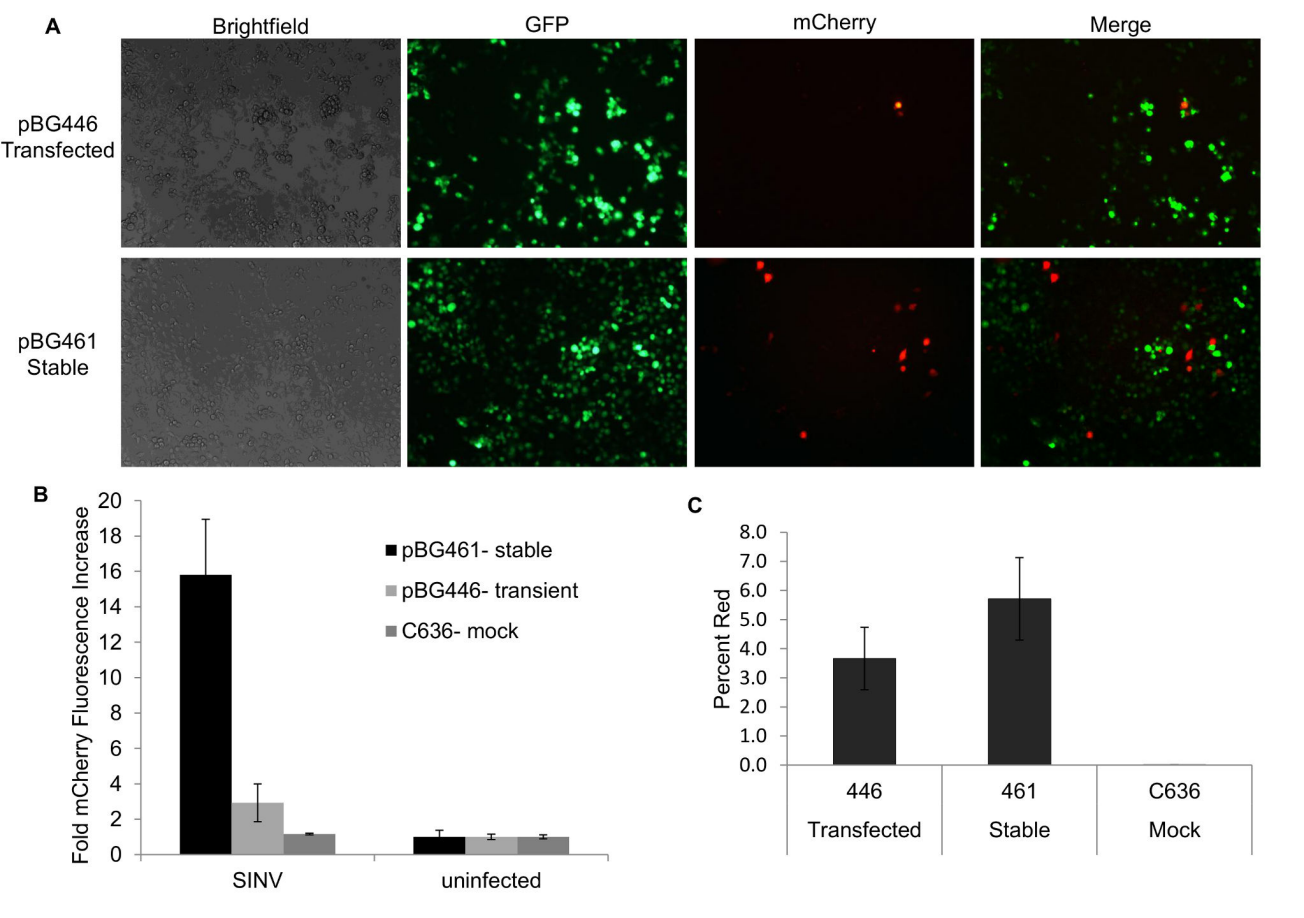
Stably Transformed C6/36 cells express reporter mCherry during SINV infection A) Brightfield, GFP, mCherry, and overlay images are shown from SINV-GFP infected C636 cells that are pBG446 transiently transfected (top) or pBG461 stably selected cells (bottom). Infected with an MOI of 10. Images collected at 48hours post infection. **B**) Average mCherry fluorescence is significantly higher in pBG461 stably transformed C6/36 cells than transiently transfected cells. **C**) Averages of total red fluorescence was calculated as a percent of total green fluorescence and is displayed for pBG446 transfected, pBG461stably selected, or C636 mock cells.

To determine if mosquito cells could be stably transformed to express alphavirus reporter RNAs, C6/36 cells were transfected with reporter constructs containing a puromycin acetyltransferase (PAC) gene (pBG461- Figure 1) and transfected cells were selected with puromycin. A bulk stable cell line was established that expressed the reporter RNA and detectable amounts of mCherry reporter protein only when infected with SINV but not in untransfected C636 or uninfected control cells (data not shown). Reporter detection was significantly higher with the stable cell line (pBG461) than transient transfection (pBG446) during SINV infection (Figure 4A and 4B). Infections were performed with a recombinant double subgenomic sindbis virus that expresses eGFP (SINV-GFP) to visually track infection (green) and confirm the reporter (red). Interestingly, cells that are not highly infected (low amounts of SINV-GFP) tend to have higher reporter mCherry expression. Although the entire stable cell line is resistant to puromycin, indicating that all cells are transformed with our construct, only 5-6% of infected cells expressed detectable amounts of mCherry (Figure 4C). This implies that there is an intricate balance of reporter RNA and infection that needs to be achieved in order for the reporter to be detected. The cell line has been maintained for over 18 months, with consistent ability to detect SINV infection (data not shown). These results indicate that reporter RNAs can be stably expressed in mosquito cells and detect alphavirus RNA replication.

### Development of Reporter RNA Expressing Transgenic *Aedes aegypti* Mosquitoes.

**Figure 5:**
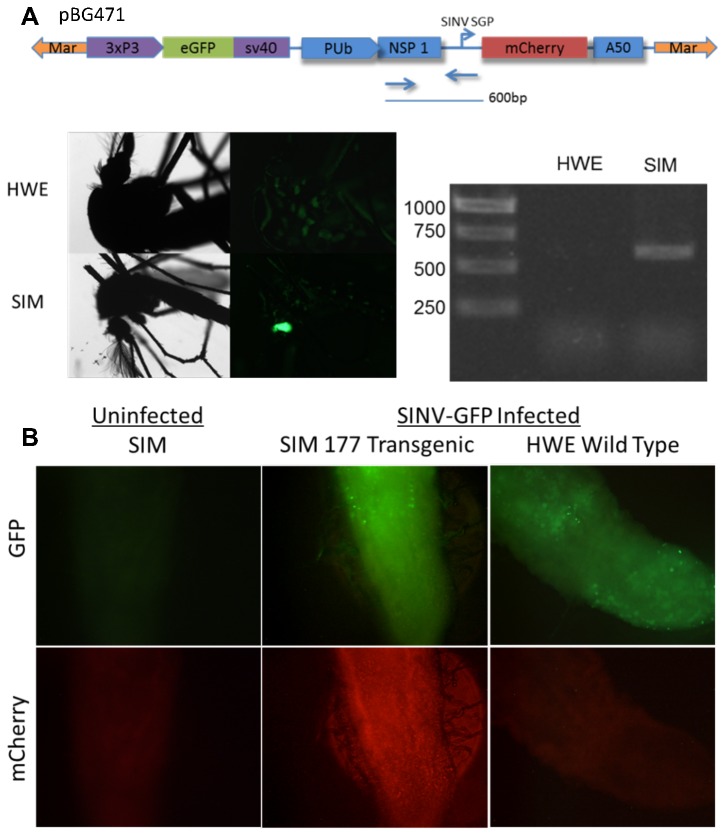
Transgenic *Aedes aegypti* express mCherry following SINV infection Verification of transgenic mosquitoes. **A**) Green eye marker for transgenesis in SIM mosquito line and Reporter DNA in transgenic mosquitoes. Genomic DNA was extracted from whole mosquitoes and used as template in PCR reaction with primers directed to the nsP1 and the SINV subgenomic promoter. The 600bp amplicon was present in SIM transgenic mosquitoes but not HWE mosquitoes. **B**) Transgenic *Ae. aegypti* expressing the mCherry reporter during SINV infection. SIM 177 transgenic family (left) and Higgs White Eye wt (right) infected with SINV-GFP are shown. 7 days post infection, images of dissected midguts with GFP (SINV) (top panels) and then the corresponding mCherry reporter expression (bottom panels).

Based on our *in vitro* results it seemed likely that this system could be used in mosquitoes to detect specific alphavirus infections. We developed transgenic mosquitoes that express SINV reporter RNAs and can express mCherry in response to infection *in vivo*. We utilized the Mariner transposon system (Mos1) to establish transgenic *Ae. aegypti* mosquitoes [41-44]. We replaced the IE3 promoter with an *Ae. aegypti* poly ubiquitin promoter (PUb) to provide more stable expression throughout the mosquito midgut than has been observed with the IE3 promoter [16]. The poly ubiquitin promoter construct (pBG471, Figure 5A) worked *in vitro* similar to the previous IE3 construct (pBG446) (data not shown). The transposon contained an eye-specific 3xp3 promoter that expressed eGFP in eyes to identify transgenic mosquitoes [22] . The reporter RNA sequence was inserted into the mariner transposon, which was co-injected with a helper plasmid expressing the mariner transposase [45] into 1,736 pre-dermoblast mosquito embryos. 327(18%) of the injected eggs were viable and hatched, were individually raised, screened for the eGFP eye marker, and outcrossed with wild type Higgs White Eye (HWE) *Ae. aegypti* mosquitoes. Each of the sindbis-induced mCherry (SIM) transgenic mosquito lines was separately hatched, screened, and females were bloodfed to maintain transgenic populations. Transgenic lines were verified by consistent eye specific eGFP expression and detection of the reporter DNA in the mosquito genomic DNA (Figure 5A). 

### Sindbis virus can activate reporter RNAs in transgenic mosquitoes

To test if Sindbis infection could activate the subgenomic reporter RNA in the transgenic lines, we infected the transgenic mosquitoes with an eGFP expressing SINV (MRE16 5’dsGFP) by feeding female mosquitoes a bloodmeal containing 7 logs of infectious virus [36,46]. Visibly engorged mosquitoes were collected after the bloodmeal and kept for 4, 7, or 14 days post-infection. At the indicated times mosquitoes were cold anesthetized and eGFP and mCherry expression was assessed under a dissecting fluorescence microscope. Mosquitoes that were visibly infected with the SINV-GFP were sacrificed and dissected midguts were imaged. We were able to detect mCherry expression in a fraction of infected transgenic mosquitoes Approximately 2-4% of the SINV-infected transgenic mosquitoes displayed detectable mCherry expression. Similar low levels of reporter fluorescence was detected in the stable C636 cell line, indicating that this system needs to be further optimized to be effective at sensitive diagnostics and modeling of infection. However, despite the low level of reporter expression, mCherry fluorescence was positively detected in bodies and midguts of a subset of mosquitoes (Figure 5B, line SIM 177 shown). mCherry expression by fluorescence or Western blot was never observed in uninfected mosquitoes, indicating that reporter RNA activation and mCherry expression was specific to SINV infection. 

## Discussion

This report describes a novel approach for detecting and monitoring Sindbis virus infection in mosquito cell culture and in live *Ae. aegypti* mosquitoes. We have developed a reporter RNA system that expresses an engineered alphavirus RNA in mosquito cells that only produces a subgenomic RNA and reporter protein in the presence of actively replicating alphaviruses. Initial work by Olivo et al. designed a reporter RNA system that expressed luciferase in BHK cells [12]. We have modified their method to be more applicable for studying alphaviruses in the natural mosquito vector and provide a way to visually track infection with a fluorescent reporter. This new system allows for rapid and simple visual detection of wild-type alphaviruses in mosquito cells. Transiently transfected or stably selected C6/36 cells expressing reporter RNAs expressed significant levels of reporter genes during infection and were only activated by related alphaviruses. Transgenic *Ae. aegypti* mosquitoes expressing a SINV reporter RNA were sensitive to replicating SINV, which triggered mCherry expression. This indicates that subgenomic reporter RNAs can diagnose alphavirus infections in live mosquitoes.

The sensitivity and quick detection of the fluorescent subgenomic reporter RNA system in cell culture provides potential applications for diagnostic tests to quickly and accurately identify virus infection. Standard diagnostics require significant time and resources to identify an infecting virus. With the subgenomic reporter RNA system, virus specific constructs (SINV, CHIKV, WEEV) could be developed that would express a visibly detectable fluorescent reporter during specific viral infections within hours and with small samples, providing a novel method for diagnosing alphavirus infections 

The species of alphavirus that activated the reporter RNAs was relatively narrow. The SINV reporter RNAs expressed significant levels of mCherry during infection by two different strains of SINV and a lower level of mCherry during CHIKV infection, but were not able to produce mCherry when infected by WEEV. SINV and CHIKV are both Old World alphaviruses, indicating some level of functional conservation between the SINV and CHIKV replicase complexes and RNA elements. New World alphaviruses such as WEEV (whose nonstructural replicase proteins are derived from Eastern Equine Encephalitis virus) were not able to induce mCherry expression, indicating that Old and New World alphavirus replicase complexes are not interchangeable. The observation that addition of a virus-specific subgenomic promoter to the SINV reporter construct did not result in mCherry expression indicates that the virus specific 5’ and 3’ UTRs are required for negative strand RNA and subgenomic RNA synthesis. A CHIKV specific reporter RNA showed similar specificity as the SINV system, demonstrating that this approach is applicable to different types of alphaviruses and that species specificity can be achieved with this approach. A potential application in future systems is to integrate multiple species-specific reporter RNAs with different fluorescent proteins into mosquito genomes that would be able to detect multiple alphavirus species in the same mosquito. Because the RNA expressed in this system is very small compared to the full-length alphavirus genome, it will prove a useful tool for dissecting the RNA and protein requirements for alphavirus RNA replication in mosquito cells in addition to its utility in identifying viral species. 

Interestingly, when we expressed reporter RNAs in C6/36 cells by transient transfection, the cells with the highest levels of eGFP expression did not express the reporter mCherry well when infected. Cells that displayed low amounts of eGFP expression (and by extension lower amounts of reporter RNA) expressed higher levels of mCherry upon infection. We have not yet determined the reason for this dichotomy, but there are several possibilities for this effect. The Sindbis virus 5’ and 3’ UTR present on the reporter RNA bind to the viral non-structural proteins, and an excess of the reporter RNA may sequester the non-structural proteins away from the full length RNA genome and reduce viral RNA replication. However, our stable cell line does not show a reduction in virus replication when compared to control C6/36 cells. Alternatively, high levels of the reporter RNA may reduce the overall level of translation in cells and reduce translation of the viral genomes to produce nonstructural proteins [47]. It may prove beneficial in subsequent versions of this system to use a less robust promoter to drive reporter RNA transcription and decrease the level of subgenomic reporter RNA. Regardless of the variability, we consistently see activation of the subgenomic promoter and expression of the reporter protein during infection in cell culture. 

Transformation of *Ae. aegypti* using the Mariner Mos1 transposase system with our reporter constructs inserted into the transposon resulted in transgenic lines that transcribe the SINV reporter RNA. We detected mCherry expression in a subset of infected transgenic mosquitoes, but did not detect mCherry expression in all infected transgenic mosquitoes. mCherry reporter expression was never detected in wild type or uninfected transgenic mosquitoes, indicating that activation of the reporter RNA was specific to virus infection. The fact that mCherry expression was detected in some but not all of the infected transgenic mosquitoes indicates that the reporter RNAs may not be active in all transgenic mosquitoes. In the stable cell lines we observed a similar effect, with some stable cells expressing mCherry during infection and others not expressing mCherry. These *in vitro* and *in vivo* findings indicate that there are mechanisms at play in mosquito cells limiting the ability of the reporter RNAs to be replicated in the presence of infecting alphaviruses. A possible mechanism for limiting the activity of the system in mosquitoes is induction of the RNA interference pathway by replication of the reporter RNA [48] [34]. The transgenic mosquitoes we present in this manuscript should be considered a proof-of-concept system that demonstrate that reporter RNA expression is a viable approach for detecting wild-type alphaviruses in mosquitoes, but this system will need to be further optimized for more efficient detection of SINV infection in live mosquitoes. Future optimization approaches will include the use of site-directed transgenesis systems, determining if RNAi affects production of reporter RNAs, and investigating different constitutive or inducible promoters, such as the *Ae aegypti* Heat Shock promoter [49] to optimize expression and minimize cellular interference. 

The reporter RNAs used in this study express fluorescent proteins during infection and allow visual confirmation of Sindbis virus infection. However, a system similar to this could be engineered to express other proteins only during active virus replication. An interesting application for this technology would be to engineer reporter RNAs that encode a cytotoxic or mosquitocidal gene, which would only be expressed concurrent with virus infection. For example, expression of the Saporin ribosomal toxin gene [50] during infection may result in abortive alphavirus infection of midgut cells and block transmission of the virus. The transgenic reporter RNA system we describe may be amenable to this approach, and we are actively examining this possibility. 

The subgenomic reporter RNA system we present can detect alphavirus infection in cell culture systems and in transgenic mosquitoes. Once optimized, this novel system will provide a valuable method to visually monitor dissemination of wild-type alphaviruses throughout insect cells and be useful for monitoring the transmission of alphaviruses from mosquitoes to mammalian hosts, increasing our understanding of viral transmission cycle. 

## References

[1] Foy BD, Myles KM, Pierro DJ, Sanchez-Vargas I, Uhlírová M et al. (2004) Development of a new Sindbis virus transducing system and its characterization in three Culicine mosquitoes and two Lepidopteran species. Insect Mol Biol 13: 89–100. doi:10.1111/j.1365-2583.2004.00464.x. PubMed: 14728670. 14728670

[2] Atkins GJ (2013) The Pathogenesis of Alphaviruses. ISRN Virology 2013: 1–22 doi:10.1128/JVI.00851-10.

[3] Weaver SC, Ferro C, Barrera R, Boshell J, Navarro J-C (2004) Venezuelan equine encephalitis. Annu Rev Entomol 49: 141–174. doi:10.1146/annurev.ento.49.061802.123422. PubMed: 14651460. 14651460

[4] Porretta D, Mastrantonio V, Bellini R, Somboon P, Urbanelli S (2012) Glacial history of a modern invader: phylogeography and species distribution modelling of the Asian tiger mosquito Aedes albopictus. PLOS ONE 7: e44515. doi:10.1371/journal.pone.0044515. PubMed: 22970238. 22970238 PMC3435282

[5] Marimoutou C, Vivier E, Oliver M, Boutin J-P, Simon F (2012) Morbidity and impaired quality of life 30 months after chikungunya infection: comparative cohort of infected and uninfected French military policemen in Reunion Island. Medicine (Baltimore) 91: 212–219. doi:10.1097/MD.0b013e318260b604. PubMed: 22732952. 22732952

[6] Phillips A, Mossel E, Sanchez-Vargas I, Foy B, Olson K (2010) Alphavirus transducing system: tools for visualizing infection in mosquito vectors. J Vis Exp: ([MedlinePgn:]) doi:10.3791/2363. PubMed: 21178952. PMC3159584 21178952

[7] Wiley MR, Roberts LO, Adelman ZN, Myles KM (2010) Double subgenomic alphaviruses expressing multiple fluorescent proteins using a Rhopalosiphum padi virus internal ribosome entry site element. PLOS ONE 5: e13924. doi:10.1371/journal.pone.0013924. PubMed: 21085714. 21085714 PMC2978087

[8] Steel JJ, Henderson BR, Lama SB, Olson KE, Geiss BJ (2011) Infectious Alphavirus Production from a Simple Plasmid Transfection. Virol J 8: 356. doi:10.1186/1743-422X-8-356. PubMed: 21771308. 21771308 PMC3156776

[9] Strauss JH, Strauss EG (1994) The alphaviruses: gene expression, replication, and evolution. Microbiology and Molecular Biology Reviews 58: 491–562. PubMed: 7968923. 10.1128/mr.58.3.491-562.1994 PMC372977 7968923

[10] Foy BD, Olson KE (2008) Alphavirus transducing systems. Adv Exp Med Biol, 627: 19–34. PubMed: 18510011. 18510011 10.1007/978-0-387-78225-6_2

[11] Nene V, Wortman JR, Lawson D, Haas B, Kodira C et al. (2007) Genome Sequence of Aedes aegypti, a Major Arbovirus Vector. Science 316: 1718–1723. doi:10.1126/science.1138878. PubMed: 17510324. 17510324 PMC2868357

[12] Olivo PD, Frolov I, Schlesinger S (1994) A cell line that expresses a reporter gene in response to infection by Sindbis virus: a prototype for detection of positive strand RNA viruses. Virology 198: 381–384. doi:10.1006/viro.1994.1046. PubMed: 8259675. 8259675

[13] Li J, Zhu W, Wang H, Li J, Zhang Q et al. (2012) Rapid, specific detection of alphaviruses from tissue cultures using a replicon-defective reporter gene assay. PLOS ONE 7: e33007. doi:10.1371/journal.pone.0033007. PubMed: 22427930. 22427930 PMC3299729

[14] Olivo PD (1996) Transgenic cell lines for detection of animal viruses. Clin Microbiol Rev 9: 321–334. PubMed: 8809463. 8809463 10.1128/cmr.9.3.321 PMC172896

[15] Jarvis DL, Fleming JA, Kovacs GR, Summers MD, Guarino LA (1990) Use of early baculovirus promoters for continuous expression and efficient processing of foreign gene products in stably transformed lepidopteran cells. Biotechnology (NY) 8: 950–955. doi:10.1038/nbt1090-950. PubMed: 1367473. 1367473

[16] Anderson MAE, Gross TL, Myles KM, Adelman ZN (2010) Validation of novel promoter sequences derived from two endogenous ubiquitin genes in transgenic Aedes aegypti. Insect Mol Biol 19: 441–449. doi:10.1111/j.1365-2583.2010.01005.x. PubMed: 20456509. 20456509 PMC3605713

[17] Hahn CS, Hahn YS, Braciale TJ, Rice CM (1992) Infectious Sindbis virus transient expression vectors for studying antigen processing and presentation. Proc Natl Acad Sci U S A 89: 2679–2683. doi:10.1073/pnas.89.7.2679. PubMed: 1372987. 1372987 PMC48725

[18] Lustig S, Jackson AC, Hahn CS, Griffin DE, Strauss EG et al. (1988) Molecular basis of Sindbis virus neurovirulence in mice. J Virol 62: 2329–2336. PubMed: 2836615. 2836615 10.1128/jvi.62.7.2329-2336.1988 PMC253389

[19] Avadhanula V, Weasner BP, Hardy GG, Kumar JP, Hardy RW (2009) A novel system for the launch of alphavirus RNA synthesis reveals a role for the Imd pathway in arthropod antiviral response. PLOS Pathog 5: e1000582. doi:10.1371/journal.ppat.1000582. 19763182 PMC2738967

[20] Geiss BJ, Shimonkevitz LH, Sackal CI, Olson KE (2007) Recombination-ready Sindbis replicon expression vectors for transgene expression. Virol J 4: 112. doi:10.1186/1743-422X-4-112. PubMed: 17963504. 17963504 PMC2164957

[21] Tsetsarkin KA, Vanlandingham DL, McGee CE, Higgs S (2007) A single mutation in chikungunya virus affects vector specificity and epidemic potential. PLoS Pathog 3: e201. doi:10.1371/journal.ppat.0030201. PubMed: 18069894. 18069894 PMC2134949

[22] Berghammer AJ, Klingler M, Wimmer EA (1999) A universal marker for transgenic insects. Nature 402: 370–371. doi:10.1038/46463. PubMed: 10586872. 10586872

[23] Pierro DJ, Powers EL, Olson KE (2008) Genetic determinants of Sindbis virus mosquito infection are associated with a highly conserved alphavirus and flavivirus envelope sequence. J Virol, 82: 2966–74. PubMed: 18160430. 18160430 10.1128/JVI.02060-07 PMC2258978

[24] Myles KM, Pierro DJ, Olson KE (2004) Comparison of the transmission potential of two genetically distinct Sindbis viruses after oral infection of Aedes aegypti (Diptera: Culicidae). J Med Entomol 41: 95–106. doi:10.1603/0022-2585-41.1.95. PubMed: 14989352. 14989352

[25] Tsetsarkin K, Higgs S, McGee CE, De Lamballerie X, Charrel RN et al. (2006) Infectious clones of Chikungunya virus (La Réunion isolate) for vector competence studies. Vector Borne Zoonotic Dis 6: 325–337. doi:10.1089/vbz.2006.6.325. PubMed: 17187566. 17187566

[26] Mossel EC, Ledermann JP, Phillips AT, Borland EM, Powers AM et al. (2013) Molecular determinants of mouse neurovirulence and mosquito infection for Western equine encephalitis virus. PLOS ONE 8: e60427. doi:10.1371/journal.pone.0060427. PubMed: 23544138. 23544138 PMC3609757

[27] Stahla-Beek HJ, April DG, Saeedi BJ, Hannah AM, Keenan SM et al. (2012) Identification of a novel antiviral inhibitor of the flavivirus guanylyltransferase enzyme. J Virol 86: 8730–8739. doi:10.1128/JVI.00384-12. PubMed: 22674988. 22674988 PMC3421717

[28] Collins TJ (2007) ImageJ for microscopy. BioTechniques 43: 25–30. doi:10.2144/000112517. PubMed: 17936939. 17936939

[29] Kim JY, Kim N, Zheng Z, Lee JE, Yenari MA (2013) The 70kD heat shock protein protects against experimental traumatic brain injury. Neurobiology of Disease. 10.1016/j.nbd.2013.06.012 PMC3799906 23816752

[30] Khoo CCH, Piper J, Sanchez-Vargas I, Olson KE, Franz AWE (2010) The RNA interference pathway affects midgut infection- and escape barriers for Sindbis virus in Aedes aegypti. BMC Microbiol 10: 130. doi:10.1186/1471-2180-10-130. PubMed: 20426860. 20426860 PMC2877022

[31] Kim SW, Li Z, Moore PS, Monaghan AP, Chang Y et al. (2010) A sensitive non-radioactive northern blot method to detect small RNAs. Nucleic Acids Res 38: e98. doi:10.1093/nar/gkp1235. PubMed: 20081203. 20081203 PMC2853138

[32] Adelman ZN, Jasinskiene N, Vally KJM, Peek C, Travanty EA et al. (2004) Formation and loss of large, unstable tandem arrays of the piggyBac transposable element in the yellow fever mosquito, Aedes aegypti. Transgenic Res 13: 411–425. doi:10.1007/s11248-004-6067-2. PubMed: 15587266. 15587266

[33] Adelman ZN, Jasinskiene N, James AA (2002) Development and applications of transgenesis in the yellow fever mosquito, Aedes aegypti. Mol Biochem Parasitol 121: 1–10. doi:10.1016/S0166-6851(02)00028-2. PubMed: 11985858. 11985858

[34] Franz AW, Sanchez-Vargas I, Adelman ZN, Blair CD, Beaty BJ et al. (2006) Engineering RNA interference-based resistance to dengue virus type 2 in genetically modified Aedes aegypti. Proc Natl Acad Sci U S A 103: 4198–4203. doi:10.1073/pnas.0600479103. PubMed: 16537508. 16537508 PMC1449670

[35] Pierro DJ, Powers EL, Olson KE (2008) Genetic determinants of Sindbis virus mosquito infection are associated with a highly conserved alphavirus and flavivirus envelope sequence. J Virol 82: 2966–2974. doi:10.1128/JVI.02060-07. PubMed: 18160430. 18160430 PMC2258978

[36] Seabaugh RC, Olson KE, Higgs S, Carlson JO, Beaty BJ (1998) Development of a chimeric sindbis virus with enhanced per Os infection of Aedes aegypti. Virology 243: 99–112. doi:10.1006/viro.1998.9034. PubMed: 9527919. 9527919

[37] Franz AWE, Sanchez-Vargas I, Piper J, Smith MR, Khoo CCH et al. (2009) Stability and loss of a virus resistance phenotype over time in transgenic mosquitoes harbouring an antiviral effector gene. Insect Mol Biol 18: 661–672. doi:10.1111/j.1365-2583.2009.00908.x. PubMed: 19754743. 19754743 PMC4839482

[38] Hardy RW, Rice CM (2005) Requirements at the 3' end of the sindbis virus genome for efficient synthesis of minus-strand. RNA - Journal of Virology 79: 4630–4639. doi:10.1128/JVI.79.8.4630-4639.2005. 15795249 PMC1069581

[39] Frolov I, Hardy R, Rice CM (2001) Cis-acting RNA elements at the 5' end of Sindbis virus genome RNA regulate minus- and plus-strand RNA synthesis. RNA 7: 1638–1651. doi:10.1017/S135583820101010X. PubMed: 11720292. 11720292 PMC1370205

[40] Weaver SC, Hagenbaugh A, Bellew LA, Netesov SV, Volchkov VE et al. (1993) A comparison of the nucleotide sequences of eastern and western equine encephalomyelitis viruses with those of other alphaviruses and related RNA viruses. Virology 197: 375–390. doi:10.1006/viro.1993.1599. PubMed: 8105605. 8105605

[41] Franz AW, Sanchez-Vargas I (2006) Engineering RNA interference-based resistance to dengue virus type 2 in genetically modified Aedes aegypti. PubMed: 16537508 10.1073/pnas.0600479103 PMC1449670 16537508

[42] Franz AWE, Jasinskiene N, Sanchez-Vargas I, Isaacs AT, Smith MR et al. (2011) Comparison of transgene expression in Aedes aegypti generated by mariner Mos1 transposition and ΦC31 site-directed recombination. Insect Mol Biol 20: 587–598. doi:10.1111/j.1365-2583.2011.01089.x. PubMed: 21699593. 21699593 PMC3556457

[43] Coates CJ, Jasinskiene N, Morgan D, Tosi LR, Beverley SM et al. (2000) Purified mariner (Mos1) transposase catalyzes the integration of marked elements into the germ-line of the yellow fever mosquito, Aedes aegypti. Insect Biochem Mol Biol 30: 1003–1008. doi:10.1016/S0965-1748(00)00110-7. PubMed: 10989286. 10989286

[44] Atkinson PW, Pinkerton AC, O'Brochta DA (2001) Genetic transformation systems in insects. Annu Rev Entomol 46: 317–346. doi:10.1146/annurev.ento.46.1.317. PubMed: 11112172. 11112172

[45] Jasinskiene N, Juhn J, James AA (2007) Microinjection of A. aegypti embryos to obtain transgenic mosquitoes. … of visualized experiments: JoVE. 10.3791/219 PMC2557089 18979017

[46] Pierro DJ, Myles KM, Foy BD, Beaty BJ, Olson KE (2003) Development of an orally infectious Sindbis virus transducing system that efficiently disseminates and expresses green fluorescent protein in Aedes aegypti. Insect Mol Biol 12: 107–116. doi:10.1046/j.1365-2583.2003.00392.x. PubMed: 12653932. 12653932

[47] Patel RK, Burnham AJ, Gebhart NN, Sokoloski KJ, Hardy RW (2013) Role for subgenomic mRNA in host translation inhibition during Sindbis virus infection of mammalian cells. Virology 441: 171–181. doi:10.1016/j.virol.2013.03.022. PubMed: 23601784. 23601784 PMC3660432

[48] Cirimotich CM, Scott JC, Phillips AT, Geiss BJ, Olson KE (2009) Suppression of RNA interference increases alphavirus replication and virus-associated mortality in Aedes aegypti mosquitoes. BMC Microbiol 9: 49. doi:10.1186/1471-2180-9-49. PubMed: 19265532. 19265532 PMC2660349

[49] Carpenetti TLG, Aryan A, Myles KM, Adelman ZN (2012) Robust heat-inducible gene expression by two endogenous hsp70-derived promoters in transgenic Aedes aegypti. Insect Mol Biol 21: 97–106. doi:10.1111/j.1365-2583.2011.01116.x. PubMed: 22142225. 22142225 PMC3259147

[50] Fabbrini MS, Rappocciolo E, Carpani D, Solinas M, Valsasina B et al. (1997) Characterization of a saporin isoform with lower ribosome-inhibiting activity. Biochem J 322 ( 3): 719–727. PubMed: 9148741. 9148741 10.1042/bj3220719 PMC1218247

